# A novel preimplantation genetic testing strategy for a subtelomeric genetic disorder: A case study

**DOI:** 10.1016/j.gendis.2023.05.013

**Published:** 2023-07-04

**Authors:** Songchang Chen, Li Wang, Luting Chen, Weihui Shi, Junyu Zhang, Yuting Hu, Yinyu Wang, Li Jin, Jianzhong Sheng, Feng Zhang, Yanting Wu, Hefeng Huang, Chenming Xu

**Affiliations:** a Obstetrics and Gynecology Hospital, Institute of Reproduction and Development, Fudan University, Shanghai 200090, China; b The International Peace Maternity and Child Health Hospital, School of Medicine, Shanghai Jiao Tong University, Shanghai 200030, China; c Research Units of Embryo Original Diseases, Chinese Academy of Medical Sciences, Shanghai 200030, China; d Key Laboratory of Reproductive Genetics (Ministry of Education), Department of Reproductive Endocrinology, Women's Hospital, Zhejiang University School of Medicine, Hangzhou, Zhejiang 310058, China; e Shanghai Key Laboratory of Embryo Original Diseases, Shanghai 200030, China; f Shanghai First Maternity and Infant Hospital, Tongji University School of Medicine, Shanghai 200030, China

Preimplantation genetic testing (PGT) is a precise and effective technique for detecting inherited pathogenic mutations to prevent birth defects. However, there are few reports on PGT for pseudo-autosomal genetic diseases. In this study, both X-linked and Y-linked short stature homeobox-containing (**SHOX**) gene mutations were identified in the Leri-Weill dyschondrosteosis (LWD) affected family, suggesting a *de novo* event of X–Y pseudo-autosomal homologous recombination during sperm meiosis in the common ancestor of the affected family members. A novel PGT strategy with sequential analysis of polar bodies and embryos (PGT-Sean) was applied to the **SHOX** mutation carriers. Following PGT-Sean, a healthy offspring was born to the LWD-affected family. For families with pseudo-autosomal genetic diseases and other genetic disorders linked to subtelomeric regions, in case of high recombination rates and lack of multiple region-specific markers, it is recommended to perform PGT-Sean to enable a more accurate clinical genetic diagnosis.

Pseudo-autosomal regions (PARs) are located at subtelomeric homology between the human X and Y chromosomes. During meiosis, PARs behave like autosomes and have a crossover rate 17-fold higher than the genome-wide average, especially in PAR1.^1^ The LWD, an inherited mesomelic dysplasia, is a pseudo-autosomal genetic disorder.^2^ The leading clinical indicators of LWD are disproportionate growth, with limb shortening, cubitus valgus, genu valgum, and Madelung's deformity. Haploinsufficiency of the short stature homeobox-containing gene (*SHOX*) is associated with short stature and the prevalence of *SHOX* mutations is high in cases of LWD.

A 26-year-old woman suffering from LWD visited the assisted reproductive technology (ART) center to conceive a healthy child. In addition, the patient was infertile for two years after her marriage. Her husband was healthy with no family history of any hereditary diseases. Moreover, semen analysis and sperm morphology assessment of her husband were normal, and karyotype analysis showed that the couple was euploid. Physical examination, pelvic examination, and gynecological ultrasound were performed. The radiographs showed that the patient's forearms were disproportionally short with carpal deformity, which was consistent with Madelung's deformity (Fig. S1). Besides, the patient's right fallopian tube was present with adhesion and partial block on hysterosalpingogram. The LWD-affected family included seven affected members and the inherited *SHOX* mutation (c.633+2T > C) was detected in all living LWD-affected members (Fig. 1). The pseudo-autosomal dominant inheritance mode of the family was neither X-linked dominant inheritance (II-1) nor X-linked recessive inheritance (III-5 and III-7). An event of XY pseudo-autosomal homologous recombination during sperm meiosis might have occurred in the LWD-affected male individual I-1, who died without biological samples being available. For the ART procedure, 13 oocytes and then 8 embryos derived from *in vitro* fertilization were obtained. Data from the embryo laboratory favor the No. 6, 7, and 9 embryos were high-quality embryos, while No. 1, 2, 3, 5, and 8 were low quality. We carried out karyomapping and haploblock summary of the pseudo-autosomal regions of human chromosomes X and Y as representative of all embryos with their first polar bodies for the proband. There was no *SHOX* mutation in the trophectoderm of No. 1, 3, 6, 7, and 9 embryos, while the *SHOX* c.633+2T > C mutation was present in the first polar body of embryos 1, 3, 7, and 9 (Fig. S2). One embryo (No. 9) without the *SHOX* c.633+2T > C variant was transferred into the patient's uterus. Four weeks after embryo transfer, a clinical gestation sac was detected by ultrasound. As an indispensable part of the PGT procedure, analysis of the amniotic fluid puncture revealed no *SHOX* c.633+2T > C mutation in the fetus. A healthy girl of 39 gestation weeks and 3285 g was delivered vaginally. In addition, genetic analysis of the umbilical cord blood validated the prenatal diagnosis (Fig. S3). One-year follow-up of the baby showed no abnormalities in growth development.

**Figure 1:**
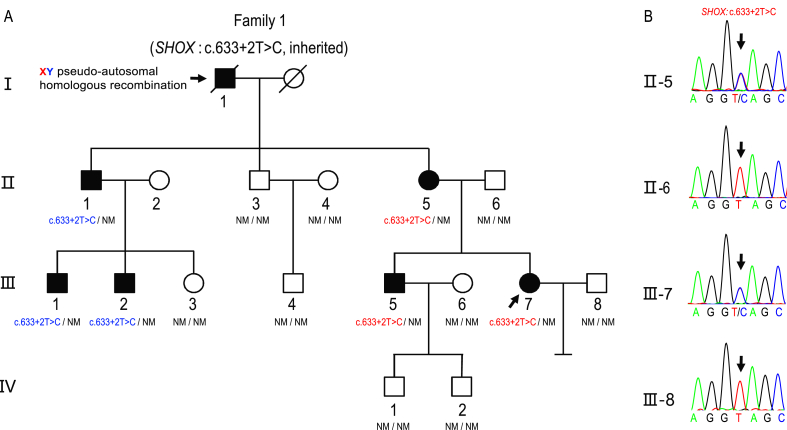
Pedigrees and inheritance of the *SHOX* gene mutation in the LWD-affected family. **(A)** Pedigrees of the LWD-affected family with an inherited *SHOX* mutation (c.633+2T > C). Squares indicate male family members, and circles indicate female family members. Solid symbols indicate affected family members and hollow symbols indicate unaffected family members. The family contains seven affected members with LWD. The arrow indicates the proband. The Y-linked *SHOX* c.633+2T > C variant is shown in blue, while the X-linked *SHOX* variant was shown in red. The mode of inheritance of the *SHOX* c.633+2T > C variant in this family suggests an XY pseudo-autosomal homologous recombination event in the LWD-affected male individual I-1. NM, no mutation. **(B)** Sanger sequencing reveals the *SHOX* c.633+2T > C only existed in the LWD-affected individuals.

The most common *SHOX* mutations are gene deletions of various sizes, and these deletions account for approximately 80% of *SHOX* mutations. In this study, both X-linked and Y-linked *SHOX* mutations were identified in this LWD-affected family, suggesting a previously unreported *de novo* event of X–Y pseudo-autosomal homologous recombination during sperm meiosis in the common ancestor (I-1).

One study found that multiplex ligation-dependent probe amplification (MLPA) was reliable for detecting aberrations involving *SHOX* while karyomapping and FISH were more effective in cases with mosaics.^3^ Karyomapping is a universal method based on the genome-wide SNP markers, allowing not only linkage analysis for monogenic disorders, but also diagnosis of chromosomal imbalances including aneuploidy, triploidy, and uniparental heterodisomy.^4^ In pilot experiments, we analyzed 136 high-frequency SNPs at the *SHOX* locus and its upstream region (chrX:0-646,823) using the karyomapping system (Table S1). The results indicated that there were only four maternal informative SNPs (rs28661974, rs6645171, rs962934, and rs35507574), and even worse there was the only one key SNP (rs28661974). To achieve accurate prenatal genetic testing for monogenic diseases (PGT-M), we used sequential analysis of polar bodies and embryos for this LWD-affected family, which had never been reported before.

The use of polar bodies in PGT, first reported in 1990,^5^ was based on the fact that the first and second polar bodies produced during maternal meiosis were predictable for the maternal contribution to the embryos. The polar body-based PGT approach has always been used to screen for chromosomal abnormalities in women with chromosomal disorders or at high risk of embryonic aneuploidies. However, the amount of DNA from polar bodies was more limited than from cleavage-stage and blastocyst biopsy, so the risk of allele dropout (ADO), amplification failure, and contamination still existed in the polar body-based PGT approach. If ADO occurs, heterozygous embryos will appear as false “homozygotes” or “compound heterozygotes”, leading to the misdiagnosis of PGT. In our case, neither the first polar body nor the trophectoderm of the NO.6 embryo carried the *SHOX* c.633+2T > C mutation, confirming the presence of ADO. Hence, the blastocyst biopsy following sequential polar body biopsies, performed in this study, ensured the PGT procedure to avoid the transfer of affected embryos.

In this study, a targeted PGT-M strategy based on sequential biopsy of polar bodies and blastocysts was performed in an LWD-affected family with the **SHOX** mutation of c.633+2T > C, which greatly improved the diagnostic efficiency of gene recombination regions. Although invasive and noninvasive prenatal diagnosis could help reduce the incidence of birth defects, the accuracy of PGT is still more important for reducing iatrogenic abortion and ethnic restrictions in some countries. For families with pseudo-autosomal genetic diseases and other genetic disorders related to subtelomeric regions, PGT with sequential analysis of polar bodies and embryos (PGT- Sean) is a new operative method to block hereditary birth defects.

## Ethics declaration

The study and PGT treatment were approved by the International Peace Maternity and Child Health Hospital Reproductive Medicine Review Board. Written informed consent was obtained from all participants prior to enrollment.

## Conflict of interests

The authors declare that they have no competing interests.

## Funding

This work was supported by the research grants from the subproject of the 10.13039/501100012166China's National Key Research and Development Program (No. 2021YFC2701002, 2022YFC2703700, 2022YFC2703702), the 10.13039/501100001809National Natural Science Foundation of China (No. 81971344, 82171677, 82192864, 82088102, 81901495), the Shanghai Municipal Commission of Science and Technology Program (China) (No. 22S31901500, 21Y21901002, 23ZR1408000), 10.13039/100017950Shanghai Municipal Health Commission (No. GW-10.1-XK07), Shanghai Municipal Commission of Health and Family Planning (China) (No. 202140110), CAMS Innovation Fund for Medical Sciences (No. 2019-I2M-5-064), Collaborative Innovation Program of Shanghai Municipal Health Commission (China) (No. 2020CXJQ01), Clinical Research Plan of SHDC (China) (No. SHDC2020CR1008A), Shanghai Clinical Research Center for Gynecological Diseases (China) (No. 22MC1940200), Shanghai Urogenital System Diseases Research Center (China) (No. 2022ZZ01012), and Shanghai Frontiers Science Research Center of Reproduction and Development (China).

## Author contributions

H.H., C.X., L.C., S.C., and Y. Wu conceived and designed the study. L.C., S.C., and W.S. drafted the manuscript. L.C., S.C., Y.H., J.Z., Y. Wang, W.S., L.W., and L.J. collected the data and analyzed and interpreted the data. L.C., S.C., and J.Z. performed the statistical analysis. H.H., C.X., F.Z., and J.S. critically revised the manuscript for important intellectual content. All authors provided administrative, technical, and material support and approved the final version of the article.

## Appendix A Supplementary data

Multimedia component 1 Multimedia component 1

Fig. S1 Radiographs demonstrating Madelung's deformity in the proband. (A) Anteroposterior radiograph of the left arm. (B) Anteroposterior radiograph of the right arm. (C) Lateral x-ray image of the left arm. (D) Lateral x-ray image of the right arm. Fig. S1

Fig. S2 Mutation analysis of first polar bodies and trophectoderm from embryos. Karyomap and haploblock summary of the pseudo-autosomal region of human chromosomes X and Y as representative of all embryos derived from *in vitro* fertilization for the proband. Maternally inherited variants appear as yellow (shared with reference) and green in karyomapping, while paternally inherited variants appear as blue (shared with reference) and red. Chromosomes are mapped according to the predicted phase by BlueFuse software, and alternating colors represent recombination events. Data from the embryo laboratory indicate that embryos 6, 7, and 9 are high-quality embryos. There is no **SHOX** mutation in the 3 trophectoderm, while the **SHOX** c.633+2T > C mutation is present only in the first polar body of embryos 7 and 9. Embryos 1, 2, 3, 5, and 8 are not high-quality embryos. There is no **SHOX** mutation in the trophectoderm of embryos 1 and 3, while the trophectoderm of embryos 2, 5, and 8 carry the **SHOX** c.633+2T > C variant. NM, no mutation. Fig. S2

Fig. S3 Mutation analysis in the proband's newborn. Sanger sequencing results are shown for the proband, her husband, and the umbilical cord blood, respectively. The proband carried the heterozygous **SHOX** mutation of c.633+2T > C, while her husband and the cord blood carried the normal alleles of **SHOX**. NM, no mutation. Fig. S3
