# Supplementary material for: A novel preimplantation genetic testing strategy for a subtelomeric genetic disorder: A case study
Source: Genes Dis. 2023 Jul 4;11(4):101014. doi: 10.1016/j.gendis.2023.05.013 (PMC10904184; doi:10.1016/j.gendis.2023.05.013)
Supplement: Multimedia component 1 [file mmc1.docx]

**Table S1 High frequency SNP loci upstream of *SHOX* gene**

| **Probe ID** | **Chromosome** | | **Position (hg19)** | | **Informative** | | **Father*** | | **Mother*** | | **Reference*** |
| --- | --- | --- | --- | --- | --- | --- | --- | --- | --- | --- | --- |
| rs28410390 | X | | 93118 | | Not informative | | NC | | NC | | NC |
| rs7471926 | X | | 161341 | | Not informative | | BB | | BB | | BB |
| rs28404746 | X | | 166168 | | Not informative | | AA | | AA | | AA |
| rs6423165 | X | | 169805 | | Not informative | | BB | | BB | | BB |
| rs6649917 | X | | 172881 | | Not informative | | AA | | AA | | AA |
| rs6644972 | X | | 178624 | | Not informative | | AA | | AA | | AA |
| rs28699277 | X | | 184508 | | Not informative | | AA | | AA | | AA |
| rs28463388 | X | | 191998 | | Not informative | | AA | | AA | | AA |
| rs28669107 | X | | 195014 | | Not informative | | BB | | BB | | BB |
| rs28811324 | X | | 200185 | | Not informative | | BB | | BB | | AB |
| rs6603172 | X | | 202439 | | Not informative | | AA | | AA | | AA |
| rs6644979 | X | | 205383 | | Not informative | | BB | | BB | | BB |
| rs28396319 | X | | 206637 | | Not informative | | NC | | NC | | NC |
| rs28661974 | X | | 210312 | | Mother informative | | AA | | AB | | AA |
| rs28667171 | X | | 213100 | | Not informative | | AA | | AA | | AA |
| rs12401269 | X | | 216845 | | Not informative | | AA | | AA | | AA |
| rs28736870 | X | | 220770 | | Not informative | | BB | | BB | | BB |
| rs28585017 | X | | 223916 | | Not informative | | AA | | AA | | AA |
| rs4074621 | X | | 230592 | | Father informative | | AB | | AA | | AA |
| rs5948792 | X | | 287573 | | Not informative | | AB | | BB | | AB |
| rs35428662 | X | | 293493 | | Not informative | | BB | | BB | | BB |
| rs2738373 | X | | 299591 | | Not informative | | BB | | BB | | BB |
| rs2738360 | X | | 302966 | | Not informative | | BB | | BB | | BB |
| rs2738349 | X | | 308565 | | Father informative | | AB | | AA | | AA |
| rs2738341 | X | | 312029 | | Not informative | | BB | | BB | | BB |
| rs2738397 | X | | 315988 | | Not informative | | BB | | BB | | BB |
| rs2738330 | X | | 317940 | | Not informative | | BB | | BB | | BB |
| rs2738319 | X | | 320203 | | Not informative | | NC | | NC | | NC |
| rs5945446 | X | | 324662 | | Not informative | | BB | | BB | | BB |
| rs5987274 | X | | 333275 | | Not informative | | AA | | AA | | AA |
| rs5987279 | X | | 336578 | | Not informative | | NC | | NC | | NC |
| rs7472503 | X | | 342775 | | Not informative | | BB | | BB | | BB |
| rs7054845 | X | | 345795 | | Not informative | | BB | | BB | | BB |
| rs28520035 | X | | 349692 | | Not informative | | AA | | AA | | AA |
| rs5950673 | X | | 353979 | | Not informative | | AB | | BB | | AB |
| rs5950677 | X | | 356282 | | Not informative | | NC | | NC | | NC |
| rs35755665 | X | | 358680 | | Not informative | | BB | | BB | | BB |
| rs5991320 | X | | 361126 | | Not informative | | AB | | AA | | AB |
| rs28727445 | X | | 365190 | | Not informative | | BB | | BB | | BB |
| rs6603256 | X | | 370446 | | Not informative | | AA | | AA | | AB |
| rs7876569 | X | | 374959 | | Not informative | | AB | | BB | | AB |
| rs7887387 | X | | 377570 | | Not informative | | BB | | BB | | BB |
| rs4986490 | X | | 381525 | | Not informative | | BB | | BB | | BB |
| rs5950823 | X | | 385562 | | Not informative | | AB | | AB | | AA |
| rs5950833 | X | | 388839 | | Father informative | | AB | | BB | | BB |
| rs6603178 | X | | 391928 | | Not informative | | AB | | AB | | NC |
| rs6645051 | X | | 394325 | | Not informative | | BB | | BB | | BB |
| rs7881207 | X | | 400108 | | Not informative | | BB | | AA | | AA |
| rs28485951 | X | | 403873 | | Not informative | | AA | | AB | | AB |
| rs6603184 | X | | 404868 | | Not informative | | BB | | AB | | AB |
| rs5991346 | X | | 407015 | | Not informative | | AA | | AA | | AA |
| rs5991186 | X | | 409910 | | Not informative | | AA | | AB | | AB |
| rs5991188 | X | | 412827 | | Not informative | | BB | | BB | | BB |
| rs5991357 | X | | 416025 | | Not informative | | AA | | BB | | BB |
| rs28680530 | X | | 419224 | | Not informative | | BB | | AA | | AA |
| rs28575158 | X | | 421498 | | Not informative | | AA | | AA | | AA |
| rs7880674 | X | 425232 | | Not informative | | | AA | AA | | AA | |
| rs28411149 | X | 428240 | | Not informative | | | BB | BB | | BB | |
| rs5991199 | X | 430311 | | Not informative | | | BB | AB | | AB | |
| rs6422431 | X | 435263 | | Not informative | | | AB | AB | | AA | |
| rs5950698 | X | 438820 | | Not informative | | | AB | AB | | BB | |
| rs5991211 | X | 443567 | | Not informative | | | BB | BB | | AB | |
| rs5991136 | X | 446601 | | Not informative | | | BB | BB | | BB | |
| rs5950631 | X | 449065 | | Not informative | | | AB | AA | | AB | |
| rs28505550 | X | 455405 | | Not informative | | | BB | BB | | BB | |
| rs34046041 | X | 457678 | | Not informative | | | BB | BB | | BB | |
| rs5991228 | X | 463631 | | Not informative | | | BB | AB | | AB | |
| rs28473635 | X | 467370 | | Not informative | | | BB | BB | | BB | |
| rs7883792 | X | 468417 | | Not informative | | | AA | AA | | AA | |
| rs28588838 | X | 472642 | | Not informative | | | BB | BB | | AB | |
| rs5991243 | X | 477422 | | Father informative | | | AB | BB | | BB | |
| rs28373840 | X | 480177 | | Not informative | | | AA | AA | | AA | |
| rs5950726 | X | 482467 | | Not informative | | | NC | NC | | NC | |
| rs5950737 | X | 487046 | | Not informative | | | BB | AB | | AB | |
| rs5991145 | X | 489911 | | Not informative | | | BB | BB | | BB | |
| rs36015925 | X | 492320 | | Not informative | | | AA | AA | | AA | |
| rs5991259 | X | 496859 | | Not informative | | | BB | BB | | BB | |
| rs4986463 | X | 500359 | | Not informative | | | NC | NC | | NC | |
| rs5991153 | X | 502934 | | Not informative | | | AA | AA | | AB | |
| rs5950751 | X | 506344 | | Not informative | | | AA | AA | | AB | |
| rs5991156 | X | 509591 | | Not informative | | | AA | AA | | AB | |
| rs5991278 | X | 513369 | | Not informative | | | AA | AA | | AB | |
| rs5991289 | X | 520151 | | Father informative | | | AB | AA | | AA | |
| rs28855771 | X | 522417 | | Father informative | | | AB | BB | | BB | |
| rs5991291 | X | 527467 | | Not informative | | | NC | NC | | NC | |
| rs5950786 | X | 535224 | | Father informative | | | AB | BB | | BB | |
| rs6645171 | X | 538059 | | Mother informative | | | BB | AB | | BB | |
| rs35701755 | X | 539631 | | Not informative | | | BB | BB | | AB | |
| rs5991302 | X | 542277 | | Not informative | | | AA | AA | | AA | |
| rs5950791 | X | 547306 | | Not informative | | | BB | BB | | BB | |
| rs17148936 | X | 549324 | | Not informative | | | AA | AA | | AA | |
| rs28597918 | X | 551114 | | Not informative | | | BB | BB | | BB | |
| rs962934 | X | 554983 | | Mother informative | | | AA | AB | | BB | |
| rs5950804 | X | 560620 | | Not informative | | | AB | AB | | BB | |
| rs28472901 | X | 564199 | | Not informative | | | AA | AA | | AA | |
| rs28520844 | X | 571377 | | Not informative | | | BB | BB | | BB | |
| rs35443706 | X | 578382 | | Not informative | | | BB | BB | | BB | |
| rs28636573 | X | 582264 | | Not informative | | | AA | BB | | BB | |
| rs35855719 | X | 588350 | | Not informative | | | AB | AB | | BB | |
| rs2239402 | X | 591926 | | Not informative | | | BB | BB | | BB | |
| rs2239403 | X | 592944 | | Father informative | | | AB | BB | | BB | |
| rs28451299 | X | 593732 | | Not informative | | | NC | NC | | NC | |
| rs35395237 | X | 594116 | | Father informative | | | AB | AA | | AA | |
| rs28475531 | X | 594144 | | Father informative | | | AB | BB | | BB | |
| rs28650799 | X | 594459 | | Not informative | | | AA | AA | | AA | |
| rs28683770 | X | 595843 | | Not informative | | | AB | AB | | AA | |
| rs2399901 | X | 596647 | | Not informative | | | BB | BB | | BB | |
| rs28453093 | X | 597612 | | Not informative | | | AB | AB | | AB | |
| rs28367488 | X | 597665 | | Not informative | | | AA | AA | | AA | |
| rs2895544 | X | 598372 | | Not informative | | | NC | NC | | NC | |
| rs964257 | X | 599506 | | Not informative | | | BB | BB | | BB | |
| rs2238843 | X | 600026 | | Not informative | | | AA | AB | | AB | |
| rs28633249 | X | 600467 | | Not informative | | | NC | NC | | NC | |
| rs35507574 | X | 606325 | | Mother informative | | | BB | AB | | BB | |
| rs28505100 | X | 607541 | | Not informative | | AA | | AB | | AB | |
| rs28634395 | X | 609254 | | Not informative | | NC | | NC | | NC | |
| rs17148916 | X | 609374 | | Not informative | | BB | | BB | | BB | |
| rs28408302 | X | 611291 | | Not informative | | BB | | BB | | BB | |
| rs34668574 | X | 611299 | | Not informative | | AB | | AB | | AB | |
| rs28619419 | X | 611324 | | Not informative | | BB | | BB | | BB | |
| rs17148914 | X | 611400 | | Not informative | | AA | | BB | | BB | |
| rs17148907 | X | 612188 | | Not informative | | AA | | AA | | AA | |
| rs2187644 | X | 612465 | | Not informative | | AB | | BB | | AB | |
| rs28579720 | X | 613567 | | Not informative | | AA | | AA | | AA | |
| rs28374757 | X | 613714 | | Not informative | | BB | | AA | | NC | |
| rs28547346 | X | 614840 | | Not informative | | BB | | AA | | AA | |
| rs28362085 | X | 616422 | | Not informative | | BB | | BB | | BB | |
| rs28631004 | X | 617998 | | Not informative | | BB | | BB | | BB | |
| rs28368483 | X | 618996 | | Not informative | | BB | | BB | | BB | |
| rs28495521 | X | 620216 | | Not informative | | BB | | BB | | BB | |
| rs28621935 | X | 622838 | | Father informative | | AB | | AA | | AA | |
| rs28430507 | X | 626158 | | Father informative | | AB | | BB | | BB | |
| rs34273382 | X | 631832 | | Not informative | | BB | | BB | | BB | |
| rs5946747 | X | 635172 | | Not informative | | NC | | NC | | NC | |
| rs7062505 | X | 638728 | | Not informative | | AA | | AA | | AA | |
| rs4911922 | X | 643862 | | Father informative | | AB | | BB | | BB | |

*Abbreviations: AA, homozygous A alleles; AB, heterozygous A and B alleles; BB, homozygous B alleles; NC, no call.
