# Supplementary figures and images for: A novel preimplantation genetic testing strategy for a subtelomeric genetic disorder: A case study
Source: Genes Dis. 2023 Jul 4;11(4):101014. doi: 10.1016/j.gendis.2023.05.013 (PMC10904184; doi:10.1016/j.gendis.2023.05.013)

**A**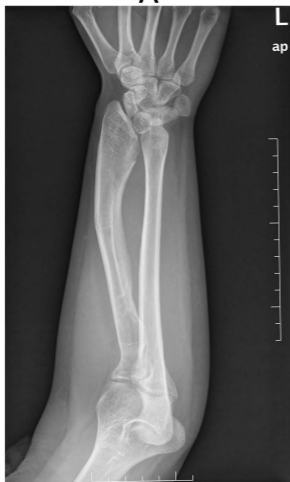**B**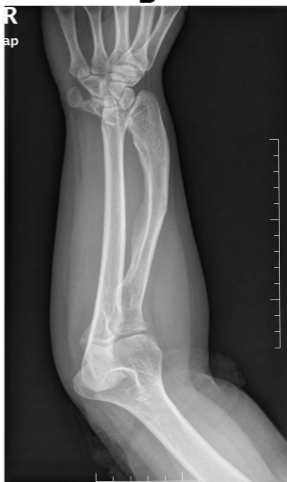**C**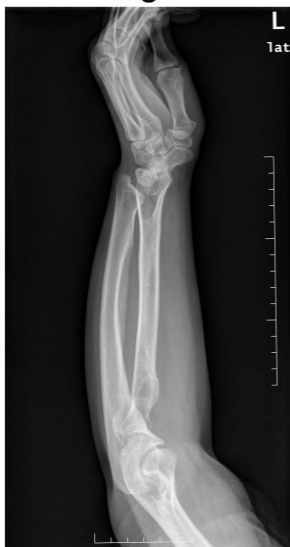**D**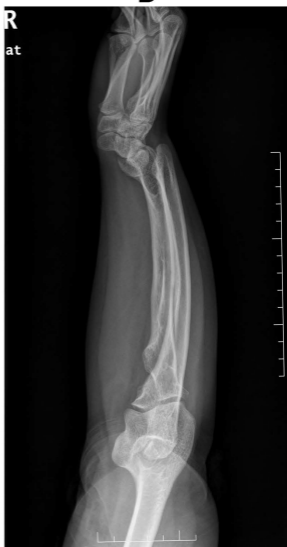

Supplement: Fig. S1 — Radiographs demonstrating Madelung's deformity in the proband. (A) Anteroposterior radiograph of the left arm. (B) Anteroposterior radiograph of the right arm. (C) Lateral x-ray image of the left arm. (D) Lateral x-ray image of the right arm. [file mmc2.pdf]

### Proband

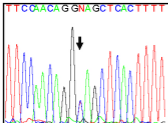

### Husband

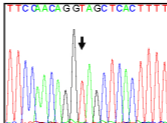

### Cord blood

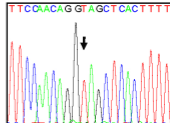

Supplement: Fig. S3 — Mutation analysis in the proband's newborn. Sanger sequencing results are shown for the proband, her husband, and the umbilical cord blood, respectively. The proband carried the heterozygous SHOX mutation of c.633+2T > C, while her husband and the cord blood carried the normal alleles of SHOX. NM, no mutation. [file mmc4.pdf]
