# Supplementary material for: A novel preimplantation genetic testing strategy for a subtelomeric genetic disorder: A case study
Source: Genes Dis. 2023 Jul 4;11(4):101014. doi: 10.1016/j.gendis.2023.05.013 (PMC10904184; doi:10.1016/j.gendis.2023.05.013)
Supplement: Fig. S2 — Mutation analysis of first polar bodies and trophectoderm from embryos. Karyomap and haploblock summary of the pseudo-autosomal region of human chromosomes X and Y as representative of all embryos derived from in vitro fertilization for the proband. Maternally inherited variants appear as yellow (shared with reference) and green in karyomapping, while paternally inherited variants appear as blue (shared with reference) and red. Chromosomes are mapped according to the predicted phase by BlueFuse software, and alternating colors represent recombination events. Data from the embryo laboratory indicate that embryos 6, 7, and 9 are high-quality embryos. There is no SHOX mutation in the 3 trophectoderm, while the SHOX c.633+2T > C mutation is present only in the first polar body of embryos 7 and 9. Embryos 1, 2, 3, 5, and 8 are not high-quality embryos. There is no SHOX mutation in the trophectoderm of embryos 1 and 3, while the trophectoderm of embryos 2, 5, and 8 carry the SHOX c.633+2T > C variant. NM, no mutation. [file mmc3.pdf]

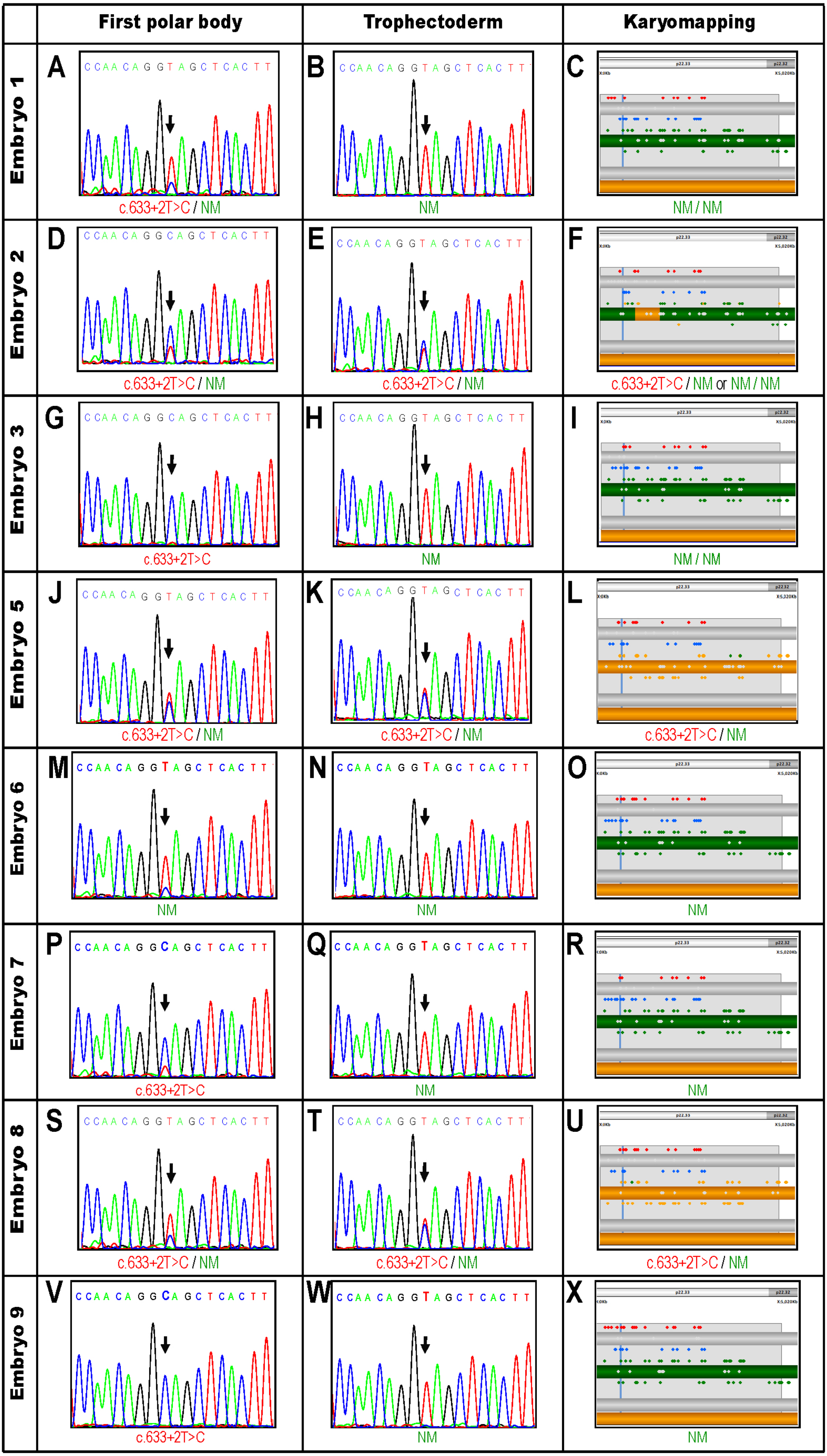
First polar body
Trophectoderm
Karyomapping
Embryo 1
A
c.633+2T>C / NM
B
NM
C
NM / NM
Embryo 2
D
c.633+2T>C / NM
E
c.633+2T>C / NM
F
c.633+2T>C / NM or NM / NM
Embryo 3
G
c.633+2T>C
H
NM
I
NM / NM
Embryo 5
J
c.633+2T>C / NM
K
c.633+2T>C / NM
L
c.633+2T>C / NM
Embryo 6
M
NM
N
NM
O
NM
Embryo 7
P
c.633+2T>C
Q
NM
R
NM
Embryo 8
S
c.633+2T>C / NM
T
c.633+2T>C / NM
U
c.633+2T>C / NM
Embryo 9
V
c.633+2T>C
W
NM
X
NM
